# Supplementary material for: Impaired bidirectional communication between interneurons and oligodendrocyte precursor cells affects social cognitive behavior
Source: Nat Commun. 2022 Mar 16;13:1394. doi: 10.1038/s41467-022-29020-1 (PMC8927409; doi:10.1038/s41467-022-29020-1)
Supplement: Supplementary file 1 — Supplementary Information [file 41467_2022_29020_MOESM1_ESM.pdf]

Supplementary information for

Impaired bidirectional communication between interneurons and oligodendrocyte precursor cells affects social cognitive behavior

Li-Pao Fang[1], Na Zhao[1], Laura C. Caudal[1], Hsin-Fang Chang[2], Renping Zhao[3], Ching-Hsin Lin[2], Nadine Hainz[4], Carola Meier[4], Bernhard Bettler[5], Wenhui Huang[1], Anja Scheller[1], Frank Kirchhoff[1,6]*, Xianshu Bai[1,]*

1. Molecular Physiology, CIPMM, University of Saarland, 66421 Homburg, Germany
2. Cellular Neurophysiology, CIPMM, University of Saarland, 66421 Homburg, Germany
3. Biophysics, CIPMM, University of Saarland, 66421 Homburg, Germany
4. Department of Anatomy and Cell Biology, University of Saarland, 66421 Homburg, Germany
5. Department of Biomedicine, University of Basel, 4056 Basel, Switzerland
6. Experimental Research Center for Normal and Pathological Aging, University of Medicine and Pharmacy of Craiova, 200349 Craiova, Romania

Correspondence to:

Prof. Dr. Frank Kirchhoff,
Molecular Physiology,
CIPMM, University of Saarland,
66421 Homburg, Germany
frank.kirchhoff@uks.eu

Dr. Xianshu Bai
Molecular Physiology,
CIPMM, University of Saarland,
66421 Homburg, Germany
xianshu.bai@uks.eu

Supplementary Methods

Oligodendrocyte specific $GABA_{B1}$ cKO mice

For oligodendrocyte specific deletion of $GABA_{B1}R$, we took advantage of TgN(PLP-Cre^{ERT2}) mice [1] crossbred with $GABA_{B1}^{lox511/lox511}$. Similarly, $PLP^{ct2/wt}$ x $GABA_{B1}R^{fl/fl}$ mice were used as cKO, while $PLP^{wt/wt}$ x $GABA_{B1}R^{fl/fl}$ or $PLP^{ct2/wt}$ x $GABA_{B1}R^{wt/wt}$ were controls after tamoxifen application.

Purity analysis of MACs-OPCs

For the purity assessment, cells were re-suspended with 1 mL "re-expression medium", seeded on the coverslips which were coated with polyornithin (P2533, Sigma). After 2 h incubation at 37 °C with 5 % CO_2, cells were processed for immunocytochemistry against PDGFRα and DAPI.

Compound action potential recordings

Compound action potentials were recorded in the corpus callosum [2]. Micropipettes with 1-3 MΩ resistance were filled with ACSF. Inward responses were evoked in current-clamp mode by varying the intensity of stimulus pulses (0.2-4.0 mA) at 1 mm distance between recording and stimulation electrodes, with a stimulus duration of 200 µs. The sample sweeps were acquired every 5 s. Conduction velocity was estimated by changing the distance from 2.5 mm to 0.5 mm between the stimulating and recording electrodes with a constant stimulus. To enhance the signal to noise ratio, we averaged at least 15 successive sweeps. Data analysis was performed with Igor pro 6.3.7.2 (WaveMetrics, Oregen, US). All the experiments were conducted at RT (22 – 24 °C).

Electron microscopy

Perfusion and dissection: Anesthetized (Ketamine/Xylazine in 0.9 % NaCl solution (all: Bayer, Leverkusen, Germany)) mice were intracardially perfused with buffered heparin (B. Braun, Melsungen, Germany) solution (end concentration 50 IU/mL) followed by perfusion fixation

with 4 % (w/v) paraformaldehyde and 0.5 % (w/v) glutaraldehyde in 0.1°M cacodylate buffer. During perfusion, the solutions were gradually cooled down from 37°C to ice cold temperatures. Whole brains were dissected, immersed in fixative solution for 24°h, and stored in 0.1°M cacodylate buffer at 4°C until further use.

Slicing: For vibratome slicing, whole brains were embedded in 10% gelatin and immersed in ice cold 0.1°M cacodylate buffer. 500 µm frontal sections were made using a vibratome (VT1000S, Leica Microsystems, Wetzlar, Germany) at a frequency of 80°Hz and 0.1°mm/s speed. Slices containing the region of interest were determined using a mouse brain atlas [3], and particular brain areas (i.e. corpus callosum) were meticulously cut out using a fine scalpel.

Embedding and examination: The specimen of the different brain regions were embedded for electron microscopy (1975) [4]. In short, samples were repeatedly rinsed in 0.1°M cacodylate buffer, osmicated in 2 % osmium tetroxide in 0.1°M cacodylate buffer for 1h, washed in distilled water, and dehydrated in an ascending series of ethanol (70 % to 100 %) and acetone (100 %, water-free). An embedding in Epon resin (EMS, Hatfield, PA, USA) followed. After thorough polymerization, semi-thin (300°nm) and ultra-thin (65nm) sections were cut using an ultra-microtome (EM UC7, Leica Microsystems, Wetzlar, Germany). Semi-thin sections were stained with Richardson blue staining solution, and examined under a light microscope (DM2500, Leica Microsystems, Wetzlar, Germany). Ultra-thin sections were contrasted with 5 % lead citrate for 5°min, examined with a transmission electron microscope (Tecnai G2, FEI, Hillsboro, OR, USA), and randomly selected areas were documented with a digital camera (MegaView III, Olympus, Shinjuku, Japan).

Supplementary figures

a

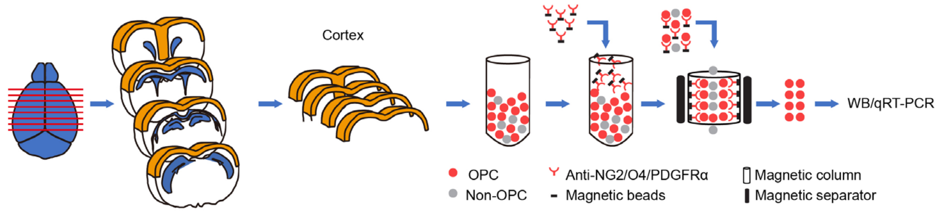

b

Mosue ID	PDGFRα$^+$	DAPI$^+$	PDGFRα$^+$ / DAPI$^+$ (%)
mouse1	50	61	81.9%
mouse2	306	348	87.9%
mouse3	384	447	85.9%
average			85.2%

c

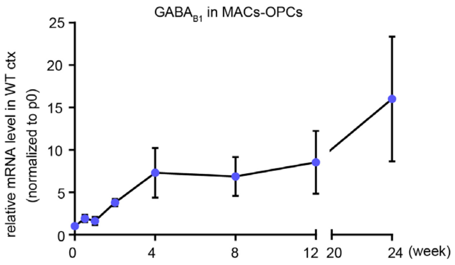

Supplementary Figure 1. GABA$_{B1}$ expression increases during cortical development. **a** Scheme of MACs (Magnetic-activated cell sorting) procedure. **b** Evaluation of OPC purity after MACs (PDGFRα$^+$/Dapi$^+$). **c** Relative mRNA levels of GABA$_B$R subunit1 (normalized to p0) increased with age (all data were acquired from C57BL/6N: p0: n=4 mice, p3: n=7 mice, p7: n=7 mice, p14: n=3 mice, 4w: n=7 mice, 8w: n=7 mice, 12w: n=4 mice, 24w: n= 3 mice). Data are shown as mean ± SEM in **c**. Source data are provided as a Source Data file.

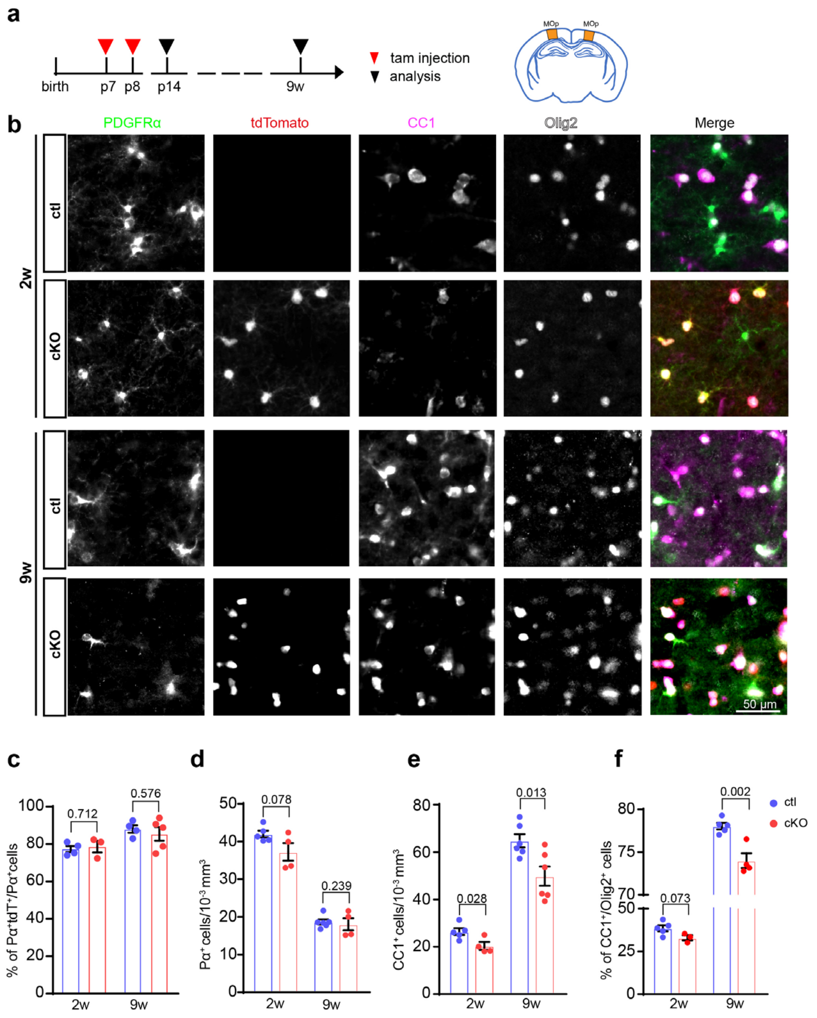

Supplementary Figure 2. OPC and oligodendrocyte density is not altered in the primary motor cortical area of mutant mice. a Experimental schedule and analyzed brain region (orange), i.e. the primary motor cortex area at the hippocampal level (MOp). **b** Cortical OPCs and OLs of ctl and cKO mice were immunolabeled with PDGFRα (Pα) and CC1, respectively, at the age of 2w and 9w. Olig2 was used as lineage marker; tdTomato expression indicates recombined cells. **c** OPC recombination efficiency in ctl and cKO MOp (2w-ctl= 77.3±1.6 (n=4 mice), 2w-cKO= 78.6±3.1 (n=3 mice), two-sided unpaired t-test; (9w-ctl= 82.9±2.0 (n=4 mice), 9w-cKO= 85.4±3.6 (n=5 mice), two-sided unpaired t-test). **d** Cell density of OPCs (Pα$^+$) in MOp of 2w- and 9w-old mice were not changed. (2w-ctl=42.0±0.9 (n=5 mice), 2w-cKO=37.2±2.3 (n=4 mice), two-sided unpaired t-test) (9w-ctl=16.6±1.0 (n=6 mice), 9w-cKO=13.5±0.7 (n=4 mice), two-sided unpaired t-test). **e** OLs (CC1$^+$) in the MOp were decreased in cKO mice at the age of 2w and 9w. (2w-ctl= 26.4±1.4 (n=5 mice), 2w-cKO=

20.4±1.6 (n=4 mice), two-sided unpaired t-test) (9w-ctl=64.8±2.8 (n=6 mice), 9w-cKO=49.8±4.1 (n=6 mice), two-sided unpaired t-test). **f** Percentage of mature OLs ($CC1^+$) of the complete lineage ($Olig2^+$) population, considered as OPC differentiation rate, was not changed at 2w and showed a slight decrease at 9w. (2w-ctl=38.5±1.7 (n=5 mice), 2w-cKO=33.0±1.5 (n=3 mice), two-sided unpaired t-test) (9w-ctl=78.0±0.4 (n=5 mice), 9w-cKO=74.0±0.9 (n=4 mice), two-sided unpaired t-test). Data are shown as mean ± SEM in **c-f**. Source data are provided as a Source Data file.

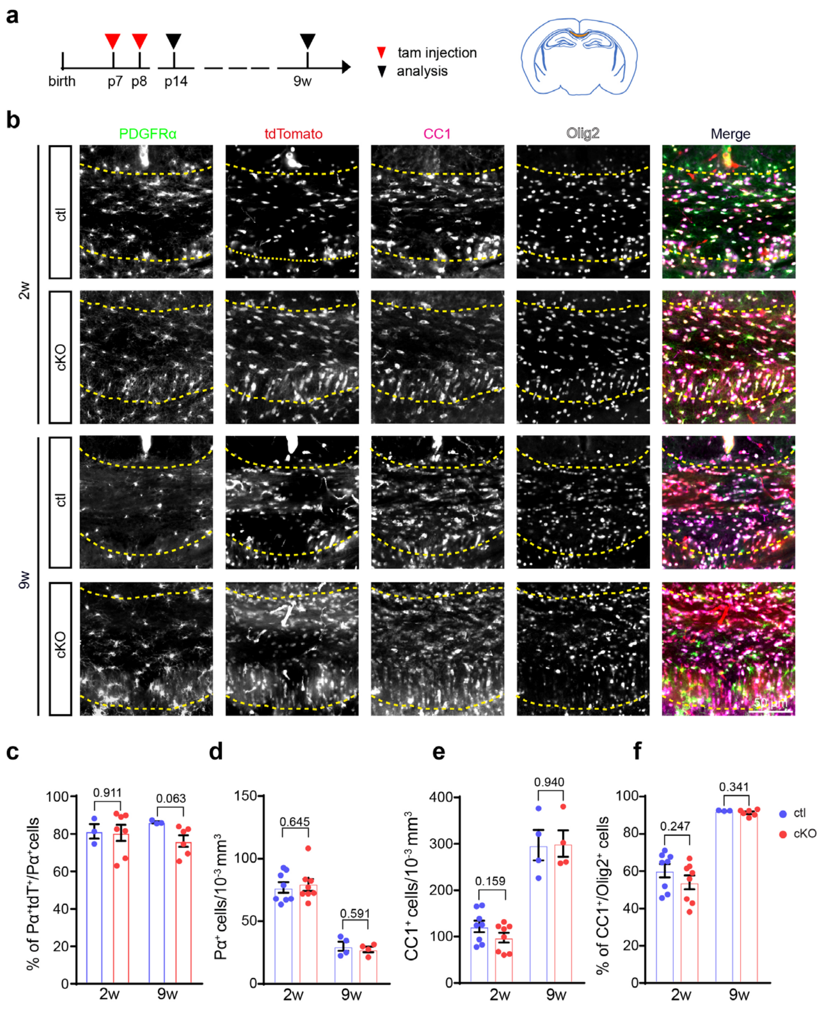

Supplementary Figure 3. OPC and oligodendrocyte density is not altered in the corpus callosum of mutant mice. a Experimental schedule and analyzed brain region (orange), i.e. corpus callosum (cc). **b** Callosal OPCs and OLs of ctl and cKO mice were immunolabeled with PDGFRα and CC1 respectively at the age of 2w and 9w. Olig2 was used as lineage marker; tdTomato expression indicates recombined cells. **c** OPC recombination efficiency in ctl and cKO cc (2w-ctl=81.4±3.9 (n=3 mice), 2w-cKO= 80.5±4.3 (n=7 mice), two-sided unpaired t-test; (9w-ctl=86.2±0.6 (n=3 mice), 9w-cKO= 76.2±3.1 (n=6 mice), two-sided unpaired t-test). **d**, **e** Cell density of OPCs (PDGFRα$^+$) and OLs (CC1$^+$) in cc of 2w- and 9w-old cKO mice were not changed. (OPC: 2w-ctl=77.0±4.2 (n=8 mice), 2w-cKO=80.0±4.7 (n=8 mice), two-sided unpaired t-test; 9w-ctl=30.0±3.7 (n=4 mice), 9w-cKO=27.6±2.3 (n=4 mice), two-sided unpaired t-test); oligodendrocytes: 2w-ctl=121.7±12.3 (n=8 mice), 2w-cKO=97.6±10.5 (n=8 mice), two-sided unpaired t-test; 9w-ctl= 296.9±32.8 (n=4 mice), 9w-cKO=300.3±28.5 (n=4

mice), two-sided unpaired t-test). **f** OPC differentiation rate was not altered at both ages. (2w-ctl=60.3±3.6 (n=8 mice), 2w-cKO=54.0±3.7 (n=8 mice), two-sided unpaired t-test) (9w-ctl=92.4±0.1 (n=3 mice), 9w-cKO=91.3±0.7 (n=6 mice), two-sided unpaired t-test). Data are shown as mean ± SEM in **c-f**. Source data are provided as a Source Data file.

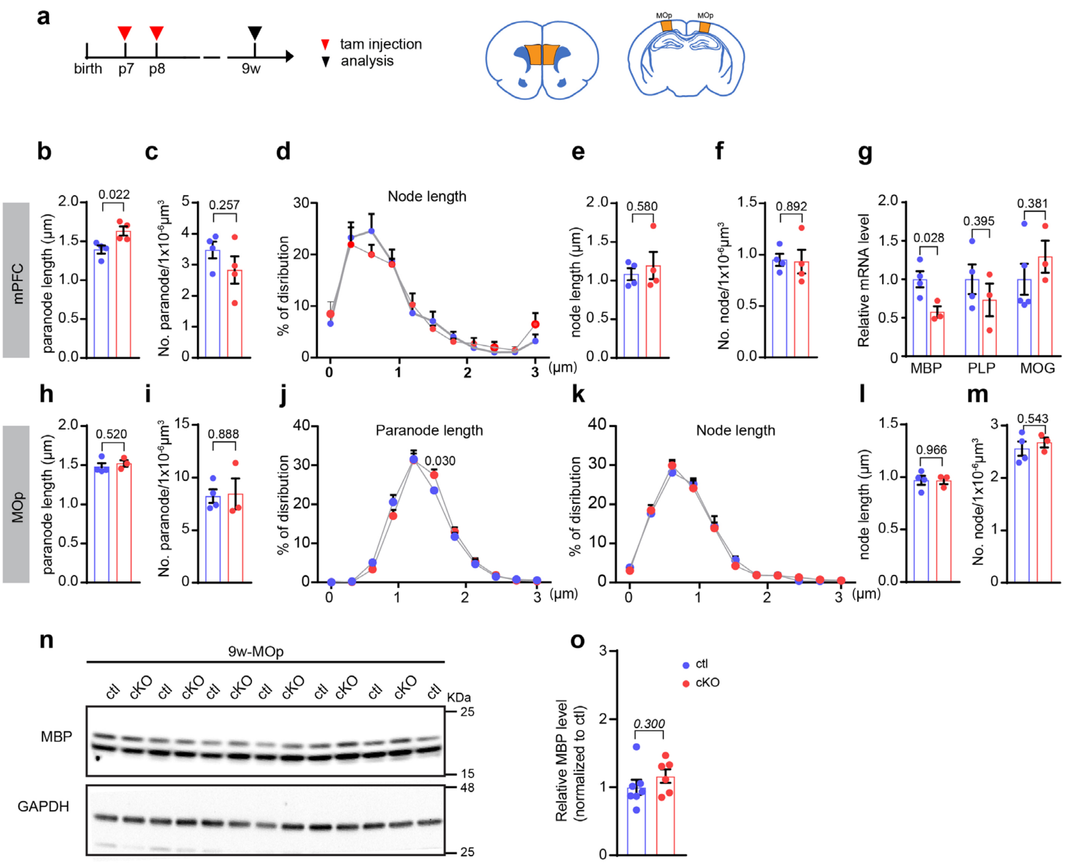

Supplementary Figure 4. Structural alterations of paranodal loops in the mPFC of mutant mice, but not in the MOp. a Scheme of experimental schedule and analyzed brain region in highlight (mPFC (medial prefrontal cortex) and MOp (primary motor cortex). **b** Paranode length was increased in the cKO mPFC. (ctl=1.4±0.1 (n=4 mice), cKO=1.6±0.1 (n=4 mice), two-sided unpaired t-test). **c** Number of paranodes remained the same in the cKO mPFC. (ctl=3. 5±0.3 (n=4 mice), cKO=2.8±0.5 (n=4 mice), two-sided unpaired t-test). **d** Quantification of node length of ctl and cKO mPFC. (ctl (n=4 mice), cKO (n=4 mice), two-sided unpaired t-test). **e**, **f** Length and number of nodes remained the same in mutant mPFC mice. Node length (ctl=1.1±0.1 (n=4 mice), cKO=0.9±0.1 (n=4 mice), two-sided unpaired t-test); Node number (ctl=0.95±0.06 (n=4 mice), cKO=0.9±0.1 (n=4 mice), two-sided unpaired t-test). **g** Relative mRNA level of MBP (myelin basic protein) was reduced in the cKO mPFC, while PLP (proteolipid protein) and MOG (myelin oligodendrocyte glycoprotein) mRNA were not affected. MBP (ctl=1±0.1 (n=4 mice), cKO=0.6±0.1 (n=3 mice), two-sided unpaired t-test); PLP (ctl=1±0.2 (n=4 mice), cKO=0.7±0.2 (n=3 mice), two-sided unpaired t-test); MOG (ctl=1±0.2

(n=5 mice), cKO=1.3±0.2 (n=3 mice), two-sided unpaired t-test). **h**, **i** Length and number of paranodes remained unchanged in mutant mice MOp. Paranode length: (ctl=1.5±0.05 (n=4 mice), cKO=1.5±0.04 (n=3 mice), two-sided unpaired t-test); Paranode number (ctl=8.2±0.6 (n=4 mice), cKO=8.5±1.5 (n=3 mice), two-sided unpaired t-test). **j**, **k** Quantification of paranode and node length of ctl and cKO MOp (ctl (n=4 mice), cKO (n=3 mice), two-sided unpaired t-test). **l**, **m** Length and number of nodes in MOp were not affected by the OPC-$GABA_BR$ deletion. Node length (ctl=1.0±0.04 (n=4 mice), cKO=1.0±0.03 (n=3 mice), two-sided unpaired t-test); Node number (ctl=2.6±0.1 (n=4 mice), cKO=2.7±0.1 (n=3 mice), two-sided unpaired t-test). **n**, **o**, MBP expression was not changed in the MOp of cKO mice. (ctl=1±0.1 (n=7 mice), cKO= 1.2±0.1 (n=6 mice), two-sided unpaired t-test). Data are shown as mean ± SEM in **b-m** and **o**. Source data are provided as a Source Data file.

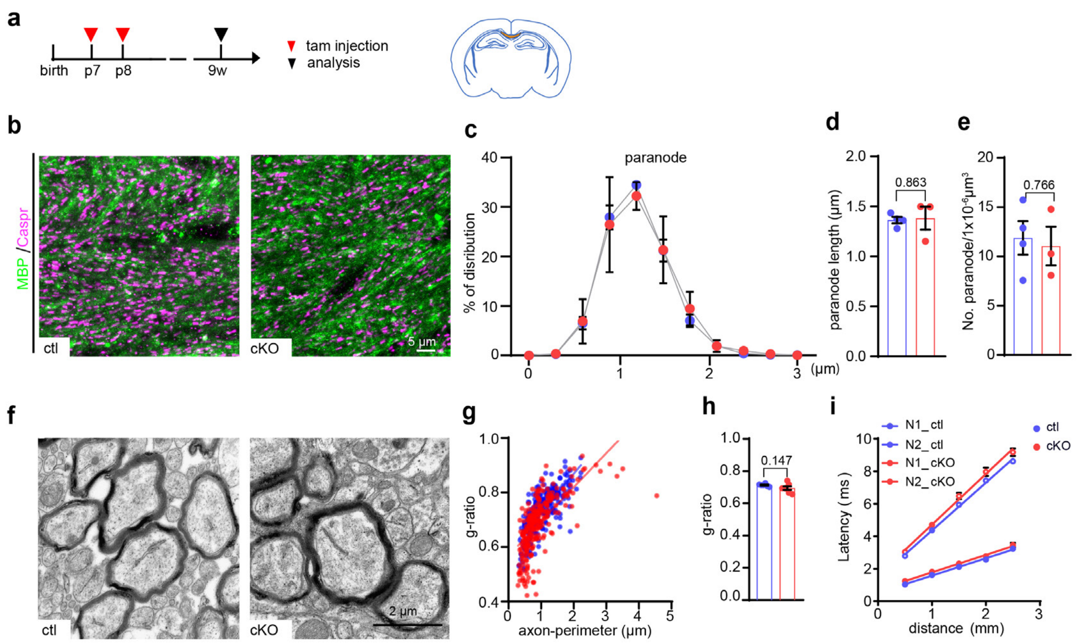

Supplementary Figure 5. Myelination remains largely unaltered in the corpus callosum of mutant mice. a Experimental schedule and analyzed brain region (orange), i.e. corpus callosum (cc). **b** Immunostaining of Caspr and MBP (myelin basic protein) in cc of ctl and cKO mice. **c** Quantification of paranode of ctl and cKO cc. (ctl (n=4 mice), cKO (n=3 mice), two-sided unpaired t-test). **d, e** Paranodal length and paranode number in ctl and cKO cc remained same. Paranodal length: (ctl=1.4±0.03 (n=4 mice), cKO=1.4±0.1 (n=3 mice), two-sided unpaired t-test); paranode number: (ctl=11.9±1.7 (n=4 mice), cKO=11.1±2.0 (n=3 mice), two-sided unpaired t-test). **f** Electron microscopic analysis of myelin ultrastructure in sagittal section of cc from ctl and cKO mice. **g**, **h** Quantification of g-ratios (the ratio between the inner and the outer diameter of the myelin sheath) did not reveal perturbations of myelin formation in cKO cc (ctl=0.7±0.001 (n=3 mice), cKO=0.7±0.01 (n=2 mice), two-sided unpaired t-test). **i** Conduction velocity of compound action potentials was not altered as indicated by extracellular field recordings. N1 indicates the conduction velocity of non-myelinated axons, N2 myelinated axons (ctl=35 cells from 3 mice; cKO=10 cells from 3 mice; two-sided unpaired t-test). Data are shown as mean ± SEM in **c-e** and **g-i**. Source data are provided as a Source Data file.

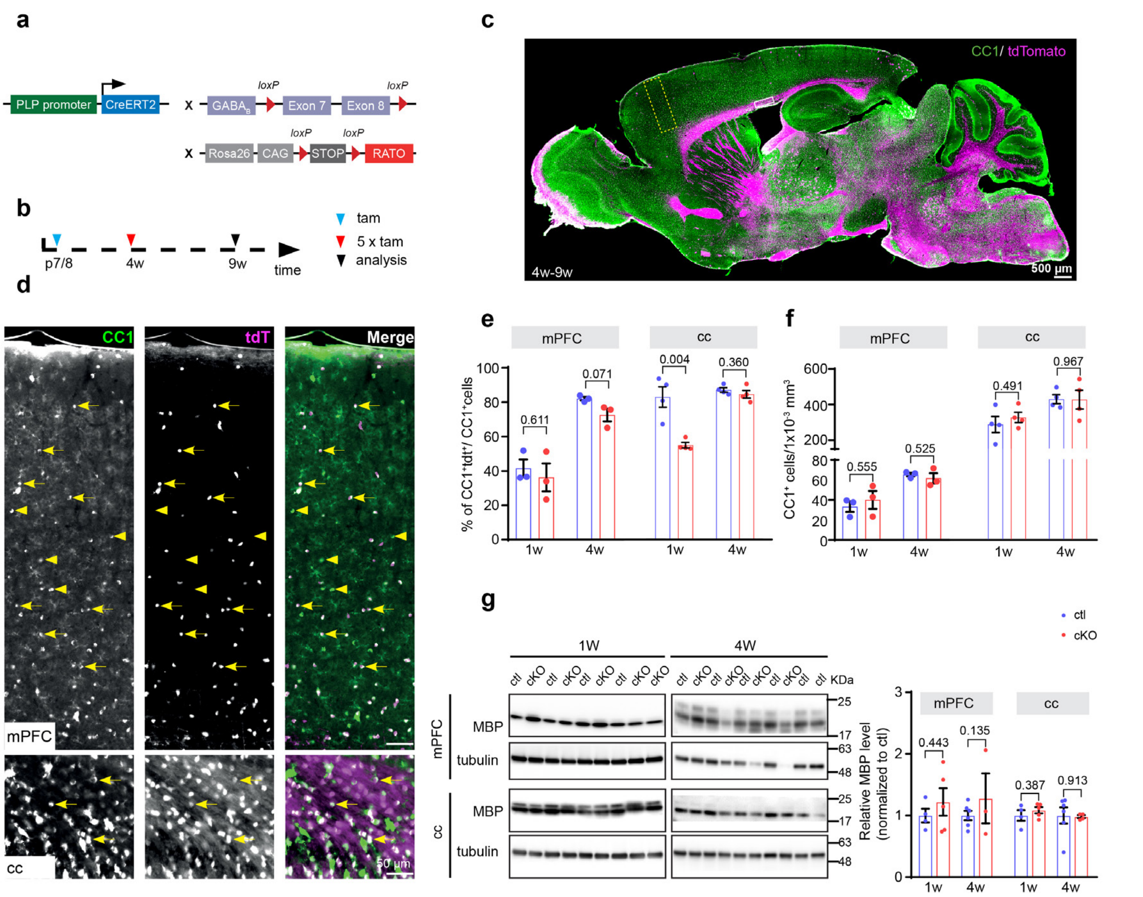

Supplementary Figure 6. Ablation of GABA$_{B1}$ in oligodendrocytes does not affect the formation of oligodendrocytes or myelin in the mPFC and corpus callosum. a Scheme of mouse lines. **b** Experimental schedule. **c** Sagittal section of CC1 immunostaining of a 9w old ctl mouse. The expression of tdTomato (tdT, magenta) indicated high recombination efficiency in numerus brain regions. **d** Magnified micrographs of the prefrontal cortex (PFC, yellow box in c) and corpus callosum (cc, white box in c). **e** Quantification of OL recombination efficiency in mPFC (medial prefrontal cortex) and cc of ctl and cKO mice. mPFC: (1w-ctl=44±7.0 (n=3 mice), 1w-cKO=32.4±3.27 (n=3 mice), two-sided unpaired t-test; 4w-ctl=84.1±2.8 (n=3 mice), 4w-cKO=77.7±4.7 (n=3 mice), two-sided unpaired t-test); cc: (1w-ctl=82.7±5.9 (n=4 mice), 1w-cKO=54.8±1.6 (n=4 mice), two-sided unpaired t-test; 4w-ctl=86.9±1.30 (n=4 mice), 4w-cKO=84.4±2.2 (n=4 mice), two-sided unpaired t-test). **f** OL density did not differ between ctl and cKO mice in both PFC and cc. arrows: recombined OL; triangles: non-recombined OL. mPFC: (1w-ctl=38.4±4.5 (n=3 mice), 1w-cKO=38.9±1.9 (n=3 mice), two-sided unpaired t-test; 4w-ctl=51.2±5.8 (n=3 mice), 4w-cKO=38.3±0.7 (n=3 mice),

two-sided unpaired t-test); cc: (1w-ctl=292.1±44.2 (n=4 mice), 1w-cKO=330.5±28.2 (n=4 mice), two-sided unpaired t-test; 4w-ctl=430.1±24.9 (n=4 mice), 4w-cKO=427.7±51.0 (n=4 mice), two-sided unpaired t-test). **g** MBP expression was identical in mPFC and cc of ctl and cKO mice, in both (1w and 4w) age groups. mPFC: (1w-ctl=1±0.1 (n=4 mice), 1w-cKO=1.2±0.2 (n=5 mice), two-sided unpaired t-test; 4w-ctl=1±0.08 (n=6 mice), 4w-cKO=1.27±0.4 (n=3 mice), two-sided unpaired t-test); cc: (1w-ctl=1±0.1 (n=4 mice), 1w-cKO=1.1±0.05 (n=5 mice), two-sided unpaired t-test; 4w-ctl=1±0.1 (n=6 mice), 4w-cKO=0.98±0.02 (n=4 mice, including one outlier, only 3 mice were analyzed), two-sided unpaired t-test). Data are shown as mean ± SEM in **e-g**. Source data are provided as a Source Data file.

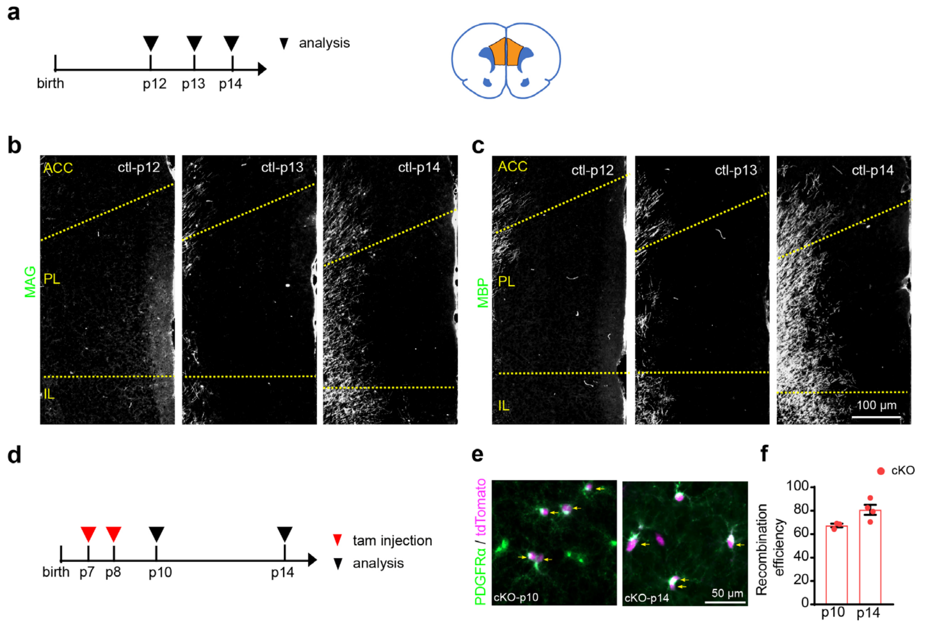

Supplementary Figure 7. Myelination starts at p14 in mPFC. a Experimental schedule and analyzed brain region (orange), i.e. mPFC (medial prefrontal cortex). **b**, **c** Immunostaining of MBP (myelin basic protein) and MOG (myelin oligodendrocyte glycoprotein) at p12-14 mPFC of ctl mice. **d** Experimental schedule for **e-f**. Immunostaining of PDGFRα^{+} combined with tdTomato (magenta) expression revealed high recombination efficiency of OPCs (% of PDGFRα^{+}tdTomato^{+}/PDGFRα^{+}) at p10 (67.4 %) and p14 (80.9 %) in the cKO mPFC. p10=67.4±1.6% (n=3 mice), p14=80.9±4.3% (n=4 mice). Data are shown as mean ± SEM in **f**. Source data are provided as a Source Data file.

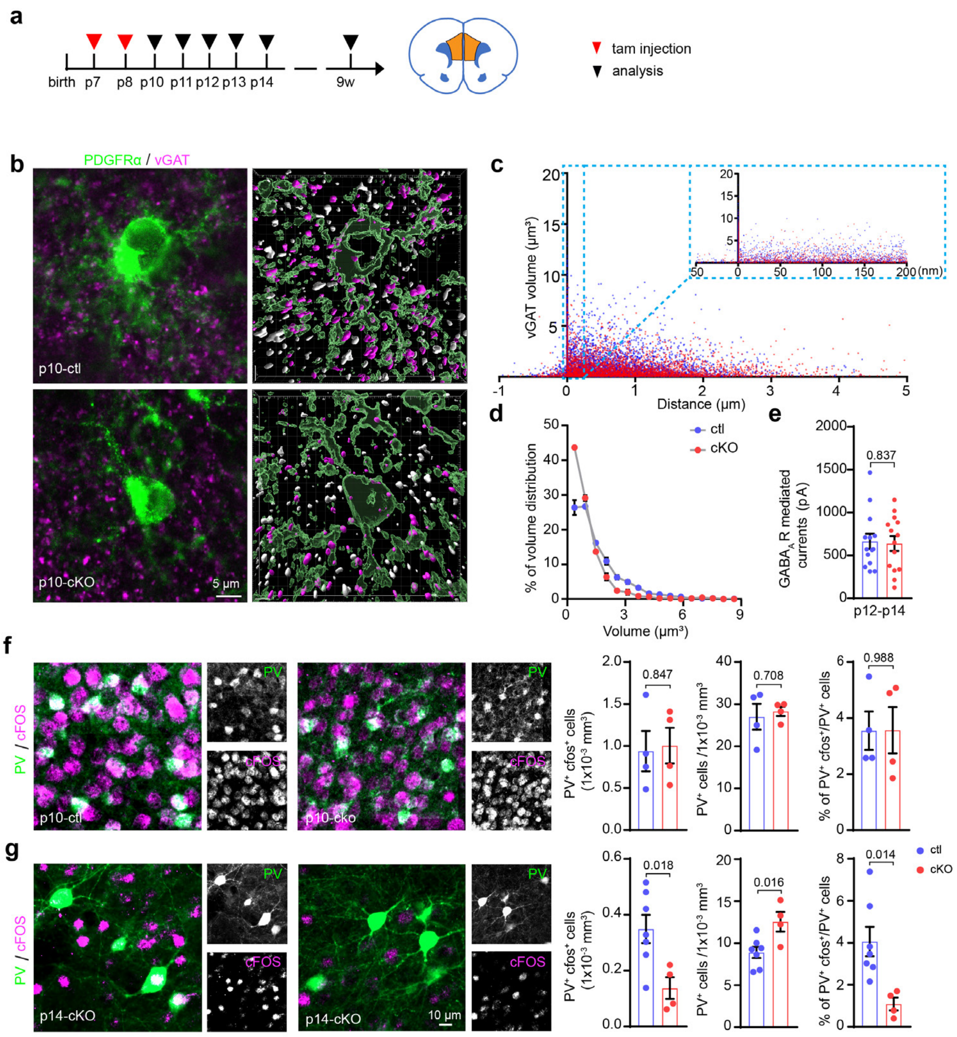

Supplementary Figure 8. At the onset of myelination, OPCs receive less GABAergic input in the mPFC of cKO mice. a Experimental schedule and analyzed brain region (orange), i.e. mPFC (medial prefrontal cortex). **b** Immunostaining of OPC (PDGFRα) and vGAT (vesicular GABA transporter) in the mPFC at postnatal day (p) 10 (micrographs and processed images of fig. 3h). **c** Quantitative analysis of vGAT puncta volume and distance to OPC surfaces. ctl=4 mice, cKO=4 mice. **d** The volume of vGAT in cKO mPFC was smaller than that in ctl group. ctl=4 mice, cKO =4 mice. **e** Electrophysiological recordings showed that $GABA_AR$ mediated currents in cKO OPCs were not perturbed during p11-14 (ctl=665.5±87.6 pA, n=14 cells from 4 mice , cKO=639.9±86.1 pA, n=14 cells from 4 mice, two-sided unpaired t-test). **f g**, Immunostaining and quantification of PV (parvalbumin) interneurons expressing c-Fos in the mPFC of p10 and p14. The PV interneuron activity is suppressed at p14 (**g**) but not

affected at p10 (**f**). p10: PV^+cFos^+ cells: (ctl=0.94±0.2 (n=4 mice), cKO=1.0±0.2 (n=4 mice); two-sided unpaired t-test); PV^+ cells: (ctl=27±3.1 (n=4 mice), cKO=28.29±1.1 (n=4 mice); two-sided unpaired t-test); % of PV^+cFos^+ cells: (ctl=3.55±0.7 (n=4 mice), cKO=3.57± 0.8 (n=4 mice); two-sided unpaired t-test). p14: PV^+cFos^+ cells: (ctl=0.35±0.05 (n=7 mice), cKO=0.14±0.04 (n=4 mice); two-sided unpaired t-test); PV^+ cells: (ctl=9.5±0.9 (n=7 mice), cKO=12.55±1.2 (n=4 mice); two-sided unpaired t-test); % of PV^+cFos^+ cells: (ctl=4.44±0.7 (n=7 mice), cKO=1.1±0.3 (n=4 mice); two-sided unpaired t-test). Data are shown as mean ± SEM in **d-g**. Source data are provided as a Source Data file.

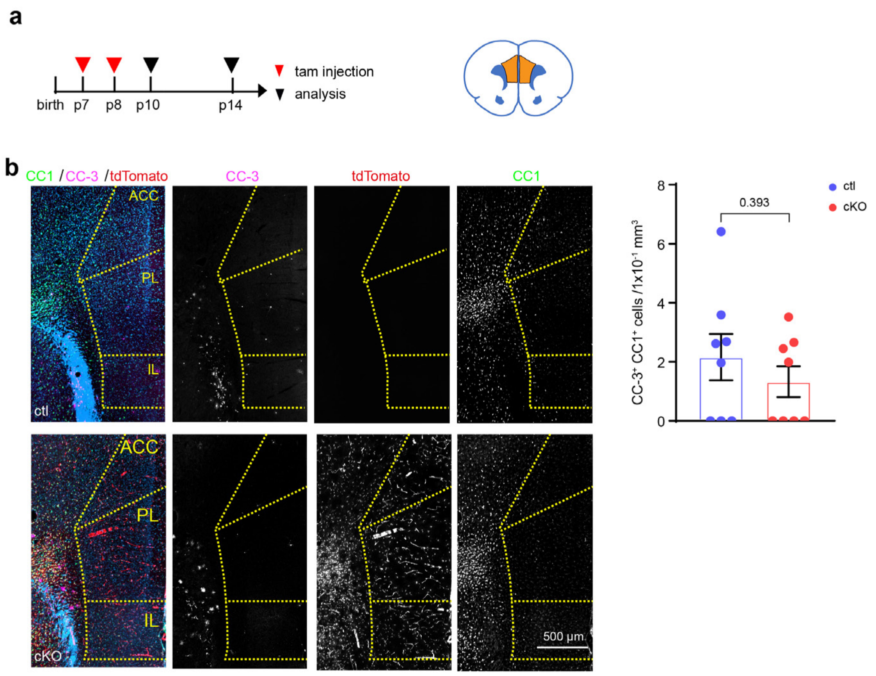

Supplementary Figure 9. Ablation of GABA$_{B1}$ in OPCs does not affect apoptosis of oligodendrocytes during development. a Experimental schedule and analyzed brain region (orange), i.e. mPFC (medial prefrontal cortex). **b** Immunostaining of OLs (CC1) and the apoptotic marker cleaved Caspase 3 (CC-3) in the mPFC of p14 mice. ctl=2.2±0.8 (n=8 mice), cKO=1.3±0.5 (n=8 mice), two-sided unpaired t-test. ACC: anterior cingulate cortex, PL=prelimbic, IL=infralimbic. Data are shown as mean ± SEM in **b**. Source data are provided as a Source Data file.

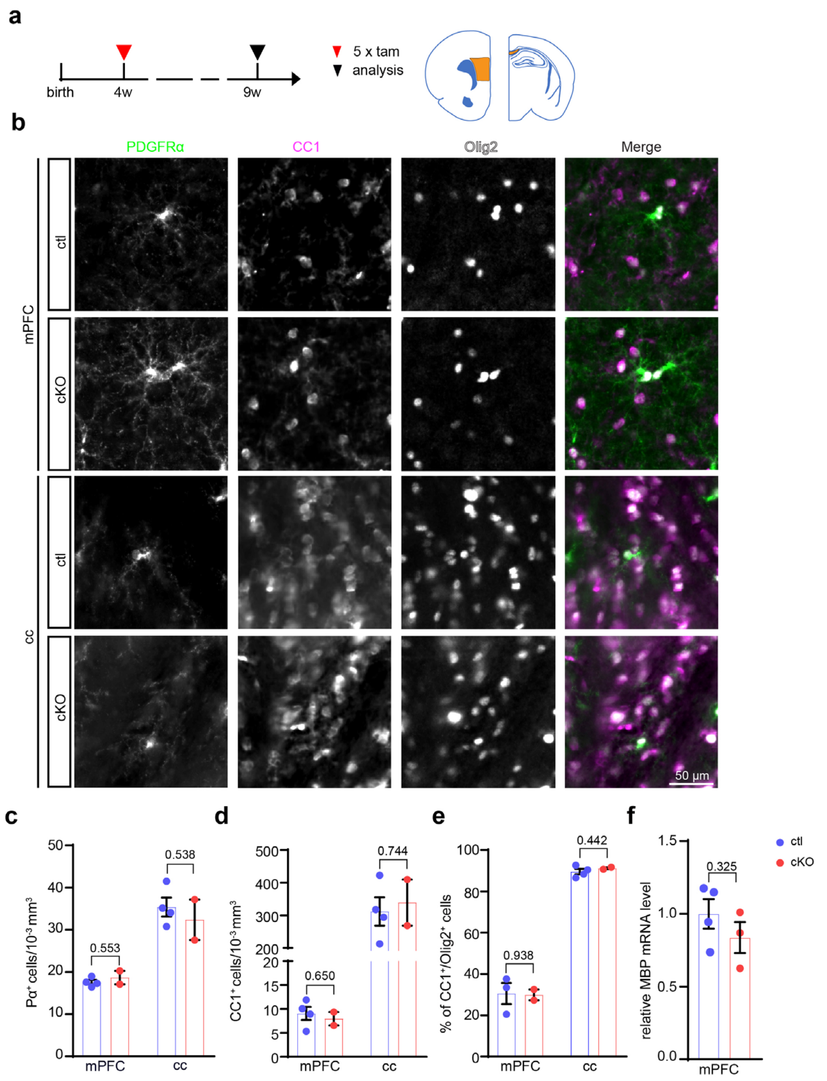

Supplementary Figure 10. Induction of OPC-GABA$_{B1}$ deletion after weaning (at 4 w of age) does not affect oligodendrogenesis. a Experimental schedule and the analyzed brain regions (mPFC (medial prefrontal cortex) and cc (corpus callosum)). **b** Immunostaining of OPCs (PDGFRα$^+$) and OLs (CC1$^+$) with Olig2 in ctl and cKO mPFC and cc at the age of 9w. **c**, **d** Cell densities of OPCs and OLs were unchanged in the mPFC and cc of ctl and cKO mice. mPFC: (OPC: (ctl=17.6±0.5 (n=4 mice), cKO=18.7±1.6 (n=2 mice), two-sided unpaired t-test)); (OL: (ctl=9.1±1.4 (n=4 mice), cKO=8.0±1.4 (n=2 mice), two-sided unpaired t-test)); cc: (OPC: (ctl=35.4±2.3 (n=4 mice), cKO=32.4±4.8 (n=2 mice), two-sided unpaired t-test)); (OL: (ctl=311.8±43.4 (n=4 mice), cKO=339.2±70.5 (n=2 mice), two-sided unpaired t-test)). **e** The proportion of OLs of all lineage cells did not change, suggesting unaltered OPC differentiation in the mutant mouse mPFC and cc. mPFC: (ctl=30.5±5.1 (n=3 mice), cKO=29.9±2.6 (n=2

mice), two-sided unpaired t-test); cc: (ctl=89.5±1.3 (n=3 mice), cKO=91.2±0.5 (n=2 mice), two-sided unpaired t-test). **f** MBP (Myelin Basic Protein expression) is not altered in the cKO mPFC at mRNA level. ctl=1±0.1 (n=4 mice), cKO=0.8±0.1 (n=3 mice), two-sided unpaired t-test). Data are shown as mean ± SEM in **c-f**. Source data are provided as a Source Data file.

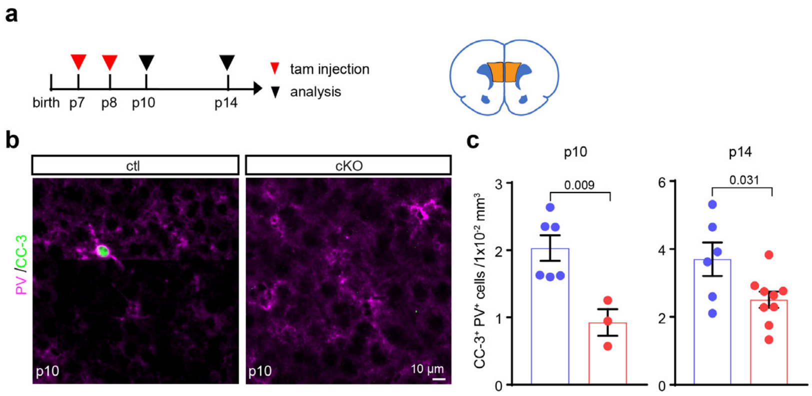

Supplementary Figure 11. Interneuron apoptosis is mitigated in the mPFC of mutant mice. a Experimental time-line and analyzed brain region (orange), mPFC (medial prefrontal cortex). **b** Immunostaining of PV (parvalbumin)$^{+}$ interneurons with the apoptotic marker (CC-3) at postnatal day (p) 10 of ctl and cKO mPFC. **c** Quantification of apoptotic PV^{+} interneurons at p10 and p14 revealed a reduced apoptosis of PV^{+} interneurons in the cKO mPFC (p10: ctl=2.0±0.5 (n=6 mice), cKO=0.9±0.3 (n=3 mice), two-sided unpaired t-test; p14: ctl=3.7±1.2 (n=6 mice), cKO=2.5±0.7 (n=9 mice), two-sided unpaired t-test). Data are shown as mean ± SEM in **c**. Source data are provided as a Source Data file.

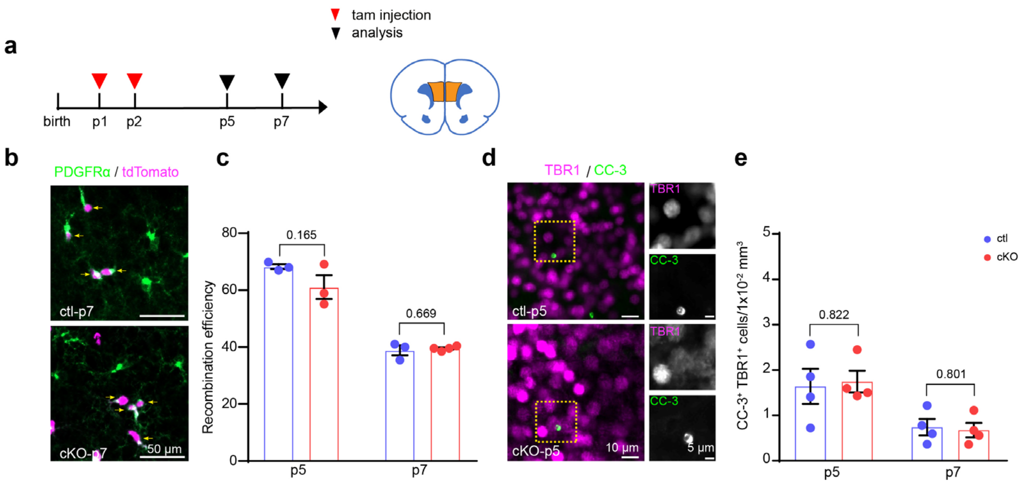

Supplementary Figure 12. Cell death or survival of excitatory neurons was not affected by early ablation of OPC-GABA$_{B1}$. a Experimental schedule and analyzed brain region (orange), mPFC (medial prefrontal cortex). **b**, **c** Immunostaining of OPCs (PDGFRα^{+}) combined with tdTomato showed about 60 % and 40 % of OPCs expressed the reporter (% of PDGFRα^{+}tdTomato^{+}/PDGFRα^{+}) at postnatal day (p) 5 and p7, respectively, both in the ctl and cKO mPFC. (p5: ctl=68.3±0.9% (n=3 mice), cKO=61±4.2% (n=3 mice); p7: ctl=38.8±1.7% (n=3 mice), cKO=39.5±1.7% (n=4 mice), two-sided unpaired t-test). **d** Immunostaining and quantification of excitatory neurons (TBR1^{+}) with the apoptotic marker CC-3 in mPFC of ctl and cKO pups at p5 (**d**) and p7 (**e**). (p5: ctl=1.6±0.4 (n=4 mice), cKO=1.7±0.2 (n=4 mice), two-sided unpaired t-test; p10: ctl=0.7±0.2 (n=4 mice), cKO=0.7±0.2 (n=4 mice), two-sided unpaired t-test). Data are shown as mean ± SEM in **c** and **e**. Source data are provided as a Source Data file.

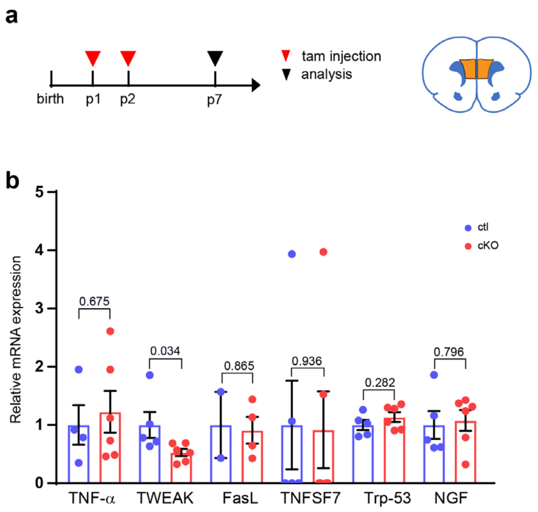

Supplementary Figure 13. TWEAK expression was down-regulated in the mPFC of OPC-GABA$_{B1}$R cKO mice. a Experimental design. **b** Relative mRNA level of cytokines in the mPFC (medial prefrontal cortex) of ctl and cKO mice at postnatal day (p) 7. (TNF-α: ctl=1±0.3 (n=4 mice), cKO=1.2±0.4 (n=6 mice), two-sided unpaired t-test); (TWEAK (TNF-like weak inducer of apoptosis): ctl=1±0.1 (n=5 mice), cKO=0.7±0.1 (n=6 mice), two-sided unpaired t-test); (FasL (Fas ligand): ctl=1±0.6 (n=2 mice), cKO=0.9±0.2 (n=4 mice), two-sided unpaired t-test); (TNFSF7 (TNF superfamily member 7): ctl=1±1.0 (n=5 mice), cKO=0.9±0.7 (n=6 mice), two-sided unpaired t-test); (Trp53 (transformation-related protein 53): ctl=1±0.1 (n=5 mice), cKO=1.1±0.1 (n=6 mice), two-sided unpaired t-test); (NGF (nerve growth factor): ctl=1±0.2 (n=5 mice), cKO=1.1±0.2 (n=6 mice), two-sided unpaired t-test). Data are shown as mean ± SEM in **b**. Source data are provided as a Source Data file.

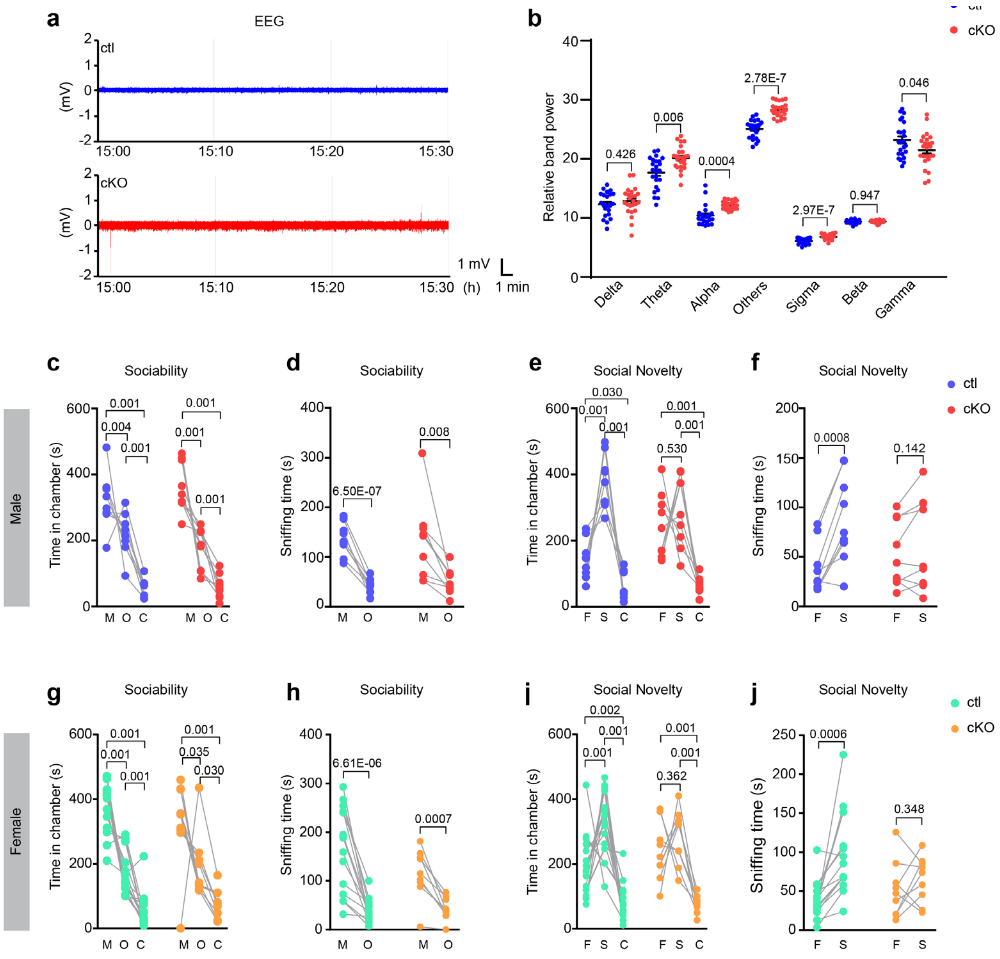

Supplementary Figure 14. OPC-GABA$_{B1}$ cKO mice exhibit altered EEGs and impaired social behavior. a, **b** EEG (electroencephalogram) recordings indicated that the power of EEG frequency bands related to cognition and sleep were altered in mutant mice, including theta, alpha and gamma (n=24 h from 4 ctl mice and 4 cKO mice). Delta: ctl=12.3±0.4, cKO=12.8±0.5; Theta: ctl=17.7±0.5, cKO=20.2±0.4; Alpha: ctl=10.4±0.3, cKO=12.2±0.1; Others: ctl=25.1±0.3, cKO=28.3±0.2; Sigma: ctl=6.1±0.1, cKO=6.8±0.1; Beta: ctl=9.4±0.06, cKO=9.4±0.05; Gamma: ctl=23.2±0.6, cKO=21.5±0.6; two-sided unpaired t-tests for individual band power. **c**, **d**, **g**, **h** Both control and mutant mice (male, **c**, **d** and female, **g h**) stayed longer in the mouse (M) chamber than in the object (O) chamber (**c**, **g**) and explored the mouse more than the object (**d**, **h**). **e**, **f**, **i**, **j** Mutant mice (male and female) did not show a preference to the familiar mouse over the stranger, suggesting impaired social cognition. (**c**: ctl: M=326±27.4s, O=219.9±21.1s, C=54.1±9.5s, (n=9 mice), Ordinary one-way ANOVA, post-hoc Tukey HSD multiple comparison; cKO: M=362.6±24.8s, O=177.8±20.8s, C=59.6±12.1s, (n=9 mice),

Ordinary one-way ANOVA, post-hoc Tukey HSD multiple comparison. **d**: ctl: M=136.5±10.9s, O=41.9±5.0s, (n=9 mice), two-sided paired t-test.; cKO: M=131.2±26.0s, O =51.5±8.6s, (n=9 mice), two-sided paired t-test. **e**: ctl: F=143.3±19.4s, S=394.9±27.9s, C=61.7±13.6s, (n=9 mice), Ordinary one-way ANOVA, post-hoc Tukey HSD multiple comparison; cKO: F=246.1±32.1s, S=289.3±35.51s, C=64.5±8.6s, (n=9 mice), Ordinary one-way ANOVA, post-hoc Tukey HSD multiple comparison. **f**: ctl: F=38.1±8.4s, S=88.34±14.66s, (n=9 mice), two-sided paired t-test; cKO: F=53.5±11.2s, S=63.8±15.5s, (n=9 mice), two-sided paired t-test. **g**: ctl: M=380.1±20.6s, O =165.7±2s, C=54.2±13.8s, (n=15 mice), Ordinary one-way ANOVA, post-hoc Tukey HSD multiple comparison; cKO: M=327.4±45.6s, O=201.1±20.8s, C=71.5±14.9s (n=9 mice), Ordinary one-way ANOVA, post-hoc Tukey HSD multiple comparison. **h**: ctl: M=165.8±22.2s, O=38.9±6.0s, (n=15 mice), two-sided paired t-test; cKO: M=115.8±17.2s, O=42.8±7.8s, (n=9 mice), two-sided paired t-test. **i**: ctl: F=197.1±13.6s, S=322.5±25.8s, C=80.4±14.6s, (n=15 mice), Ordinary one-way ANOVA, post-hoc Tukey HSD multiple comparison; cKO: F=237.3±29.8s, S=285.1±28.3s, C=77.6±9.2s, (n=9 mice), Ordinary one-way ANOVA, post-hoc Tukey HSD multiple comparison. **j**, ctl: F=38.1±6.1s, S=98.9±13.6s (n=15 mice), two-sided paired t-test; cKO: F=51.6±12.0s, S=65.4±9.8s, (n=9 mice), two-sided paired t-test. Data are shown as mean ± SEM in **b-j**. Source data are provided as a Source Data file.

Supplementary Table 1: Primary antibodies used for immunostaining

Antibodies	Host	Dilutions	Cat.No.	Company	RRID
PDGFRα	goat	1:500	AF1062	R&D Systems	AB_2236897
adenomatous polyposis coli clone 1(CC1)	mouse	1:200	OP80	Calbiochem	AB_2057371
Olig2	rabbit	1:500	AB9610	Millipore	AB_570666
MBP	mouse	1:500	SMI99	Biolegend	AB_10120130
MOG	goat		Ab115597	Abcam	AB_10898950
Neurofilament marker	mouse		SMI312	Biolegend	AB_2566782
Parvalbumin	rabbit	1:1000	PV 25	Swant	AB_10000344
Parvalbumin	mouse	1:500	P3088	Sigma	AB_477329
Caspr	rabbit	1:500	ab34151	abcam	AB_869934
DsRed	rabbit	1:1000	632496	Clontec	AB_10013483
BrdU	rat	1:1000	ab6326	abcam	AB_305426
Cleaved Caspase-3	rabbit	1:200	9661	Cell Signaling Technology	AB_2341188
Cleaved Caspase-3	mouse	1:100	STJ9744	St. John's Laboratory	AB_2341188
GAD67	mouse	1:500	MAB5406	Millipore	AB_2278725
NeuN	mouse	1:500	MAB377	Millipore	AB_2298772
CTIP2	rat	1:200	650601	Biolegend	AB_10896795
TBR1	rabbit	1:500,	49661	Cell Signaling Technology	AB_2799364
vGAT	mouse	1:500	13002	Synaptic Systems	AB_887871
FN14	mouse	1:50	314108	Biolegend	AB_2810477

Supplementary Table 2: Secondary antibodies used for immunostaining

Antibodies	Fluorophore	Cat. No.	Company
Donkey anti-mouse	Alexa Fluor® 488	A21202	Thermo Fisher
	Alexa Fluor® 546	A10036	
	Alexa Fluor® 647	A31571	
	DyLight® 755	SA5-10171	Invitrogen
Donkey anti-rabbit	Alexa Fluor® 488	A21206	Thermo Fisher
	Alexa Fluor® 546	A10040	
	Alexa Fluor® 647	A31573	
	Alexa Fluor® 790	A11374	
Donkey anti-goat	Alexa Fluor® 488	A11055	Thermo Fisher
	Alexa Fluor® 546	A11056	
	Alexa Fluor® 647	A21447	
	Alexa Fluor® 750	ab175744	abcam
Donkey anti-rat	DyLight-755	SA5-10031	Thermo Fisher
General stain for nuclei	DAPI (25 ng/ml)	A10010010	Biochimica

Secondary antibodies were diluted 1:1000 in blocking buffer.

Supplementary Table 3. Primary antibodies used for Western blot

Antibodies	Host	Dilutions	Cat.No.	Company	RRID
$GABA_{B1}R$	mouse	1:500	ab55051	abcam	AB_941703
GAPDH	mouse	1:2000	G8795	Sigma	AB_1078991
MBP	mouse	1:1000	SMI99	Biolegend	AB_10120130
Tubulin-α	rabbit	1:2000	T6074	Sigma	AB_477582

Supplementary Table 4. Primers used for cDNA qRT-PCR

Gene	Forward	Reverse
$GABA_{B1}R$	5'-CGAAGCATTTCCAACATGAC-3'	5'-CAAGGCCCAGATAGCATCATA-3'
β-actin	5'-CTTCCTCCCTGGAGAAGAGC-3'	5'-ATGCCACAGGATTCCATACC-3'
TNF-α	5’-GACGTGGAACTGGCAGAAGAG-3’	5’-TTGGTGGTTTGTGAGTGTGAG-3’
TWEAK	5’-CCGCCAGATTGGGGAATTTAC-3’	5’-AGTCCAAAGTAGGTTAGGAAGGG-3’
FasL	5’-TCCGTGAGTTCACCAACCAAA-3’	5’-GGGGGTTCCCTGTTAAATGGG-3’
TNFSF7	5’-TGTAGCGGACTACTCAGTAAGC-3’	5’-TGGGGTCCTTCCGAGGAAC-3’
Trp53	5’-CTCTCCCCCGCAAAAGAAAAA-3’	5’-CGGAACATCTCGAAGCGTTTA-3’
NGF	5’-TGATCGGCGTACAGGCAGA-3’	5’-GCTGAAGTTTAGTCCAGTGGG-3’

Supplementary References

1. Leone, D.P., *et al.* Tamoxifen-inducible glia-specific Cre mice for somatic mutagenesis in oligodendrocytes and Schwann cells. *Mol Cell Neurosci* **22**, 430-440 (2003).
2. Crawford, D.K., Mangiardi, M. & Tiwari-Woodruff, S.K. Assaying the functional effects of demyelination and remyelination: revisiting field potential recordings. *J Neurosci Methods* **182**, 25-33 (2009).
3. Franklin, K. & Paxinos, G. *The mouse brain in stereotaxic coordinate* (Academic press, 2007).
4. Glauert, A.M. *Fixation, Dehydration and Embedding of Biological Specimens. Practical methods in electron microscopy.* (North‐Holland Publishing Company, 1975).
