# Supplementary material for: Impaired bidirectional communication between interneurons and oligodendrocyte precursor cells affects social cognitive behavior
Source: Nat Commun. 2022 Mar 16;13:1394. doi: 10.1038/s41467-022-29020-1 (PMC8927409; doi:10.1038/s41467-022-29020-1)
Supplement: Supplementary file 3 — Reporting Summary [file 41467_2022_29020_MOESM3_ESM.pdf]

nature portfolio

Corresponding author(s): Frank Kirchhoff, Xianshu Bai

Last updated by author(s): Feb 17, 2022

Reporting Summary

Nature Portfolio wishes to improve the reproducibility of the work that we publish. This form provides structure for consistency and transparency in reporting. For further information on Nature Portfolio policies, see our Editorial Policies and the Editorial Policy Checklist.

Statistics

For all statistical analyses, confirm that the following items are present in the figure legend, table legend, main text, or Methods section.

n/a	Confirmed	
☐	☒	The exact sample size (*n*) for each experimental group/condition, given as a discrete number and unit of measurement
☐	☒	A statement on whether measurements were taken from distinct samples or whether the same sample was measured repeatedly
☐	☒	The statistical test(s) used AND whether they are one- or two-sided<br>*Only common tests should be described solely by name; describe more complex techniques in the Methods section.*
☒	☐	A description of all covariates tested
☐	☒	A description of any assumptions or corrections, such as tests of normality and adjustment for multiple comparisons
☐	☒	A full description of the statistical parameters including central tendency (e.g. means) or other basic estimates (e.g. regression coefficient) AND variation (e.g. standard deviation) or associated estimates of uncertainty (e.g. confidence intervals)
☐	☒	For null hypothesis testing, the test statistic (e.g. *F*, *t*, *r*) with confidence intervals, effect sizes, degrees of freedom and *P* value noted<br>*Give P values as exact values whenever suitable.*
☒	☐	For Bayesian analysis, information on the choice of priors and Markov chain Monte Carlo settings
☒	☐	For hierarchical and complex designs, identification of the appropriate level for tests and full reporting of outcomes
☒	☐	Estimates of effect sizes (e.g. Cohen's *d*, Pearson's *r*), indicating how they were calculated

Our web collection on statistics for biologists contains articles on many of the points above.

Software and code

Policy information about availability of computer code

Data collection	Image acquisition: Zeiss Axio Scan.Z1 for overview images, Zeiss LSM 710 for Caspr/MBP and vGAT/PDGFRα images, LSM 780 for PV/SMI 312/MOG images; ChemiDoc-MP (Bio-Rad) for Western blot.<br>qRT-PCR: CFX96 (Bio-Rad).<br>Nest Building: Huawei P30 lite smartphone.<br>Open field test, new object recognition test and three chamber social behavior test: USB Webcam Camera.<br>EEG recording: DSI PhysioTel RPC-1 (Data Sciences International).<br>Electrophysiology: Zeiss Axioskop 2 FS mot, QuantEM 512SC camera (Photometrics), EPC 10 USB amplifier (HEKA), Patchmaster software (v2x90.5, HEKA)
Data analysis	Image analysis: Zeiss Zen Blue 3.0 for cell counting, Imaris 9.6 for PV/SMI312/MOG and vGAT/Pα images and FIJI for Western blot.<br>Open field test: EthoVision XT 11.5 (Noldus Technology).<br>EEG analysis: Neuroscore software (Version 3.3.1, Data Sciences International).<br>Electrophysiology: Matlab 2019a, sigTOOL (Lidierth, 2009, PMID: 19056423) and custom code (https://github.com/XianshuBai/OPC-GABABR-interneuron).<br>Statistical analysis: Graphpad Prism 8.0.1

For manuscripts utilizing custom algorithms or software that are central to the research but not yet described in published literature, software must be made available to editors and reviewers. We strongly encourage code deposition in a community repository (e.g. GitHub). See the Nature Portfolio guidelines for submitting code & software for further information.

Data

Policy information about availability of data

All manuscripts must include a data availability statement. This statement should provide the following information, where applicable:

- Accession codes, unique identifiers, or web links for publicly available datasets
- A description of any restrictions on data availability
- For clinical datasets or third party data, please ensure that the statement adheres to our policy

Data Availability: Source data are provided with this paper. Further data to support the findings can be obtained upon request to the corresponding authors.
Code Availability: Custom codes used for electrophysiology analysis are available at GitHub (https://github.com/XianshuBai/OPC-GABABR-interneuron).

Field-specific reporting

Please select the one below that is the best fit for your research. If you are not sure, read the appropriate sections before making your selection.

☒ Life sciences ☐ Behavioural & social sciences ☐ Ecological, evolutionary & environmental sciences

For a reference copy of the document with all sections, see nature.com/documents/nr-reporting-summary-flat.pdf

Life sciences study design

All studies must disclose on these points even when the disclosure is negative.

Sample size	No statistical methods were used to pre-determine the sample size. For the mouse experiments, each mouse was considered as single independent sample. We have utilized all the mice available without bias. For primary cell cultures, each mouse cortex was prepared and plated into a single 24-well plate and considered as a single independent experiment. For the Oli-neu cell line experiments, cells from the same passage were considered as one experiment. Each group had at least 3 independent samples for statistical analysis. The exact sample size is indicated in each figure and figure legend.
Data exclusions	Data were preanalyzed for outliers with Rout (Q=1%) in GraphPad Prism 8.0.1. The outliers were excluded from the statistical analysis. In Supplementary Fig.6g, 4w-mPFC cKO group, a single data point was excluded from the statistical analysis due to insufficient recognition of the tubulin band.
Replication	For in vivo experiments, littermates from different breeding pairs were studied and the data were pooled. For the behavioral analysis, no replication was performed. For all immunostainings, four brain hemispheres from randomly selected brain slices of each mouse were studied. In addition, for the analysis of vGAT and PV/SMI312/MOG, at least 6 OPCs or 8 regions of interest per mouse were quantified. For the in vitro study, three independent primary cell preparations or 6 independent experiments of the Oli-neu cell line were performed. In PV/CC-3 immunostaining experiments, for each group two replicates were employed. All in vivo and vitro attempts were successful.
Randomization	For in vivo experiments, littermates with different genotypes were assigned to corresponding groups, i.e. ctl and cKO groups. For in vitro experiments, all the cells were from the same source (same passage or the same preparation) and randomly distributed to different experimental groups prior to the treatment.
Blinding	The analyses of cell counting, Imaris analysis, behavioral studies, and in vitro experiments were performed in a blind manner. The scientists analyzing the data were not informed about the groups. For Western blot and qRT-PCR analysis, blinding was not necessary since the values were analyzed by the gray intensity of bands or CT values, respectively.

Reporting for specific materials, systems and methods

We require information from authors about some types of materials, experimental systems and methods used in many studies. Here, indicate whether each material, system or method listed is relevant to your study. If you are not sure if a list item applies to your research, read the appropriate section before selecting a response.

Materials & experimental systems

n/a	Involved in the study	
☐	☒	Antibodies
☐	☒	Eukaryotic cell lines
☒	☐	Palaeontology and archaeology
☐	☒	Animals and other organisms
☒	☐	Human research participants
☒	☐	Clinical data
☒	☐	Dual use research of concern

Methods

n/a	Involved in the study	
☒	☐	ChIP-seq
☒	☐	Flow cytometry
☒	☐	MRI-based neuroimaging

Antibodies

Antibodies used	Primary antibodies: goat-anti-PDGFRα(R&D Systems, AF1062), mouse-anti-adenomatous polyposis coli clone 1(CC1) (Calbiochem, OP80), rabbit-anti-Olig2 (Millipore, AB9610), mouse-anti-MBP (Biolegend, SMI99), goat-anti-MOG (Abcam, Ab115597), mouse-anti-Neurofilament (Biolengend, SMI312), rabbit-anti-Parvalbumin (Swant, PV25), mouse-anti-Parvalbumin (Sigma, P3088), rabbit-anti-Caspr (Abcam, Ab34151), rabbit-anti-DsRed (Clontec, 632496), rat-anti-BrdU (Abcam, Ab6326), rabbit-anti-Cleaved Caspase-3 (Cell Signaling Technology, 9661), mouse-anti-Cleaved Caspase-3 (St. John's Laboratory, STJ9744), mouse-anti-GAD67 (Millipore, MAB5406), mouse-anti-NeuN (Millipore, MAB377), rat-anti-CTIP2 (Biolegend, 650601), rabbit-anti-TBR1 (Cell Signaling Technology, 49661), mouse-anti-vGAT (Synaptic Systems, 13002), mouse-anti-TWEAKR (Biolegend, 314108), mouse-anti-GABAB1 subunit (Abcam, Ab55051), mouse-anti-GAPDH (Sigma, G8795), rabbit-anti-Tubulin-α (Sigma, T6074). Secondary antibodies: Donkey anti-mouse Alexa Fluor 488 (A21202, Thermo Fisher), Alexa Fluor 546 (A10036, Thermo Fisher), Alexa Fluor 647 (A31571, Thermo Fisher), DyLight 755 (SA5-10171, Invitrogen); Donkey-anti-rabbit Alexa Fluor 488 (A21206, Thermo Fisher), Alexa Fluor 546 (A10040, Thermo Fisher), Alexa Fluor 647 (A31573, Thermo Fisher), Alexa Fluor 790 (A11374, Thermo Fisher); Donkey-anti-goat Alexa Fluor 488 (A11055, Thermo Fisher), Alexa Fluor 546 (A11056, Thermo Fisher), Alexa Fluor 647 (A21447, Thermo Fisher), Alexa Fluor 790 (Ab175744, Abcam); Donkey-anti-rat DyLight 755 (SA5-10031, Thermo Fisher); HRP anti-mouse (A9044, Sigma), HRP anti-rabbit (111-035-045, Dianova).
Validation	The primary antibodies have been validated by manufacturers and our own observations were in line with the validation.

Eukaryotic cell lines

Policy information about cell lines

Cell line source(s)	Oli-neu cell line, kindly provided by Prof. Jacqueline Trotter, University of Mainz
Authentication	Jung, M., et al. Lines of murine oligodendroglial precursor cells immortalized by an activated neu tyrosine kinase show distinct degrees of interaction with axons in vitro and in vivo. Eur J Neurosci 7, 1245-1265 (1995).
Mycoplasma contamination	The cell line was not tested for mycoplasma contamination, but no indication of contamination was observed.
Commonly misidentified lines (See ICLAC register)	N/A

Animals and other organisms

Policy information about studies involving animals; ARRIVE guidelines recommended for reporting animal research

Laboratory animals	In addition to C57BL6N wildtype mice, we have employed transgenic mice TgH(NG2-CreERT2), GABAB1lox511/lox511, Rosa26-flSTOPfl-tdTomato and TgN(PLP-CreERT2) mice maintained in C57BL6/N background. Both female and male mice were investigated in the study. The age of the mice included postnatal day 0, 3, 5, 7, 10-14, 9 weeks, 12 weeks and 24 weeks. Ages were indicated in figures and figure legends. All mice were housed at the animal facility of the CIPMM and fed with breeding diet (V1125, Sniff) ad libitum. Mice were maintained in a 12 hour light/dark cycle at 20°C with humidity at 55-70%.
Wild animals	This study did not involve wild animals.
Field-collected samples	The study did not involve samples collected from the field.
Ethics oversight	Animal husbandry and procedures were performed at the animal facility of CIPMM, University of Saarland according to European and German guidelines for the welfare of experimental animals. Animal experiments were approved by the Saarland state's ''Landesamt für Gesundheit und Verbraucherschutz" in Saarbrücken/Germany (animal license number: 65/2013, 12/2014, 34/2016, 36/2016, 03/2021 and 08/2021).

Note that full information on the approval of the study protocol must also be provided in the manuscript.
